# Human Leukocyte Antigen (HLA) Class I Down-Regulation by Human Immunodeficiency Virus Type 1 Negative Factor (HIV-1 Nef): What Might We Learn From Natural Sequence Variants?

**DOI:** 10.3390/v4091711

**Published:** 2012-09-24

**Authors:** Philip Mwimanzi, Tristan J. Markle, Takamasa Ueno, Mark A. Brockman

**Affiliations:** 1 Department of Molecular Biology and Biochemistry, Simon Fraser University, 8888 University Drive, Burnaby, British Columbia V5A 1S6, Canada; Email: pwimanzi@sfu.ca (P.M.); tmarkle@sfu.ca (T.J.M.); 2 Center for AIDS Research, Kumamoto University, 2-2-1 Honjo, Chuo-ku, Kumamoto 860-0811, Japan; Email: uenotaka@kumamoto-u.ac.jp; 3 Faculty of Health Sciences, Simon Fraser University, 8888 University Drive, Burnaby, British Columbia V5A 1S6, Canada

**Keywords:** HIV-1, Nef, immune evasion, HLA class I, cytotoxic T lymphocyte, viral sequence diversity, host immune selection pressure

## Abstract

HIV-1 causes a chronic infection in humans that is characterized by high plasma viremia, progressive loss of CD4+ T lymphocytes, and severe immunodeficiency resulting in opportunistic disease and AIDS. Viral persistence is mediated in part by the ability of the Nef protein to down-regulate HLA molecules on the infected cell surface, thereby allowing HIV-1 to evade recognition by antiviral CD8+ T lymphocytes. Extensive research has been conducted on Nef to determine protein domains that are required for its immune evasion activities and to identify critical cellular co-factors, and our mechanistic understanding of this process is becoming more complete. This review highlights our current knowledge of Nef-mediated HLA class I down-regulation and places this work in the context of naturally occurring sequence variation in this protein. We argue that efforts to fully understand the critical role of Nef for HIV-1 pathogenesis will require greater analysis of patient-derived sequences to elucidate subtle differences in immune evasion activity that may alter clinical outcome.

## 1. Introduction

This review discusses our current knowledge of the Human immunodeficiency virus type 1 (HIV-1) Negative Factor (Nef) protein and its ability to mediate immune evasion through down-regulation of Human Leukocyte Antigen class I (HLA-I) molecules on the surface of virus-infected cells. We highlight recent evidence based on cellular, molecular, and structural biology studies that extends our mechanistic understanding of this important Nef activity. Furthermore, the relevance of naturally occurring Nef sequence variation and it potential impact on protein function and clinical outcome is presented. We emphasize the critical need to examine Nef function using patient-derived viral sequences in order to fully understand the role that Nef plays in HIV-1 pathogenesis. 

### 1.1. HIV-1 Infection and Therapy

HIV-1 causes a life-long infection that is characterized by the rapid destruction of gut lymphoid compartments [1,2,3] followed by the progressive loss of peripheral blood CD4+ T lymphocytes [4]. The ultimate outcome of infection is a severe decline of the host immune system that is observed clinically as the inability to control opportunistic pathogen infections–a condition that is commonly referred to as acquired immunodeficiency syndrome (AIDS) [4]. Over the past 30 years, a large number of antiviral drugs have been developed that target essential stages in the HIV-1 replication cycle, including enzymatic processes (e.g., reverse transcriptase, protease, and integrase inhibitors) and critical viral:host protein interactions (e.g., CCR5 co-receptor antagonists) [5]. Advances in highly active antiretroviral therapy (HAART) have significantly reduced the burden of HIV/AIDS in regions of the world where these potent drug combinations are available, and HAART is currently our best option to control the spread of HIV [6,7]; however, establishment of latent viral reservoirs and ongoing low-level viremia in treated individuals require that therapy be continued for life. An improved understanding of the mechanisms that allow HIV-1 to persist and cause disease in its human host may uncover new opportunities for clinical or therapeutic intervention [8].

### 1.2. The Nef Protein

The HIV-1 accessory protein Nef is necessary for viral pathogenesis and progression to AIDS. Its *in vivo* role was first illustrated in the rhesus macaque model system where a *nef*-deleted strain of simian immunodeficiency virus (SIV) exhibited reduced viral replication, lower plasma viremia, and attenuated pathogenicity [9]. *Nef* gene deletion has also been associated with non-progressive HIV-1 infection [10,11]. Several reports have attempted to correlate Nef sequence polymorphisms with clinical outcome [12,13], with mixed results; however, relatively few studies have assessed potential functional impairment of Nef in the context of progressive or non-progressive HIV-1 infection using patient-derived sequences [14,15,16,17], and each of these reports examined only a small number of individuals.

HIV-1 Nef is a ~27kd protein that is expressed abundantly during the early stages of viral replication [18]. Nef displays diverse *in vitro* functions, including the ability to modulate a number of cell surface proteins [19], augment viral infectivity, and enhance viral replication capacity [20,21]. Down-regulation of host cell CD4 [22,23] and HLA-I [24,25] surface molecules are the most extensively studied of Nef’s activities, although some of its functions may share overlapping mechanisms. For example, Nef CD4 down-regulation activity correlates with its ability to enhance viral pathogenesis [26,27]; and lower CD4 expression on virus-infected cells may directly increase viral infectivity [28], virion release [29], viral replication [30], or prevent superinfection [31,32,33]. Although Nef’s contributions to HIV-1 pathogenesis remain incompletely understood, it has been proposed that progressive disease may require a combination of Nef-mediated functions acting at different times during the infection course [34,35].

### 1.3. HIV-1 Immune Evasion Strategies

HIV-1 evades host cellular immune responses through Nef-dependent and Nef-independent mechanisms. Nef-mediated down-regulation of HLA-I protects virus-infected cells from recognition by CD8+ T lymphocytes [36], but modulation of other host cell proteins, including CD4, CD8ß, CD28, CD74 (invariant chain), and HLA class II, may also contribute to Nef-dependent immune evasion [19,37,38]. Nef-independent immune evasion relies on the generation of viral sequence polymorphisms (“escape mutations”) within or near targeted epitopes, resulting in directional evolution of the virus away from immune selection pressure [39,40]. Despite these evasion strategies, CTL may retain antiviral activity, particularly if they recognized viral epitopes that can be presented prior to Nef-induced HLA-I down-regulation [41]. Nef selectively modulates HLA-A and HLA-B alleles through a shared sequence (Y_320_SQAASS_326_) located in their cytoplasmic tail [42,43], leaving HLA-C allele expression unchanged on the cell surface presumably to counter the innate Natural Killer cell response against HLA-devoid cells [44]. Recent data, however, suggests that Nef-mediated down-regulation of HLA-B is less robust than that of HLA-A [45], which may in part explain the observation that HLA-B alleles tend to be more protective against HIV-1 disease progression *in vivo* [46].

## 2. HLA Class I-Mediated Control of HIV-1

### 2.1. Role of HLA-I in Viral Infection

**Figure 1:**
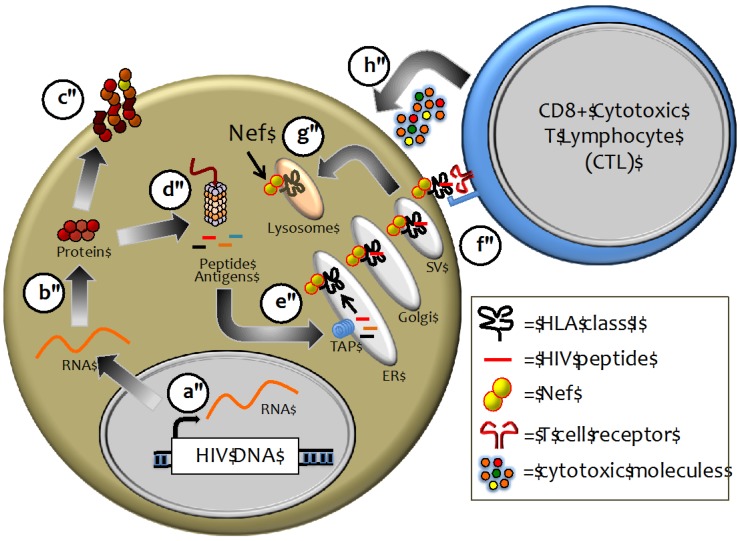
Presentation of viral peptide antigens by Human Leukocyte Antigen (HLA) class I. Human immunodeficiency virus type 1 (HIV-1) proviral gene expression, including RNA transcription (**a**) and protein translation (**b**); generates functional viral proteins (**c**) as well as truncated or mis-folded proteins that are degraded by the cellular proteasome complex to form short antigenic peptides (**d**); These peptides are transported from the cytoplasm into the endoplasmic reticulum (ER) (**e**) where they can be loaded onto HLA-I molecules. Peptide/HLA complexes traffic from the ER through the Golgi and secretory vesicle (SV) network to the plasma cell membrane, where the peptide antigens are presented to circulating cytotoxic T lymphocytes (CTL) (**f**); The viral Nef protein shuttles HLA molecules located at the cell surface or within the *trans*-Golgi network into lysosomal compartments (**g**); where they are degraded. In the absence of Nef-mediated HLA down-regulation, antigen-specific CTL respond to stimulation by releasing cytotoxic molecules, including perforin and granzymes, resulting in elimination of the virus-infected cell (h).

During the course of viral infection, the cellular proteasome complex degrades viral proteins to produce immunogenic peptide antigens. These cytosolic peptides are transported into the endoplasmic reticulum (ER), captured by HLA-I proteins, and traffic to the cell surface for presentation to circulating antiviral CD8+ cytotoxic T lymphocytes (CTL) (Figure 1). Antigen-specific T cell receptors (TCR) allow a subset of CTL to recognize these “non-self” peptides bound to HLA on the infected cell surface. Following TCR engagement with its HLA/peptide ligand, the CTL forms an “immunological synapse” with the target cell and releases antiviral cytokines and cytotoxic molecules, including perforin and granzymes, to eliminate the infected cell [47].

The critical role of HLA-I in control of infection is illustrated by the variety of strategies that viruses have independently developed in order to evade HLA-dependent immune responses [40,48,49,50]. In the case of HIV-1, Nef down-regulates HLA-A and B alleles on the cell surface and thereby reduces viral peptide presentation to CTL [19,49]. HIV-1 sequences also undergo mutational escape in an HLA-restricted manner, yielding epitope variants that are incompletely or improperly processed, presented and/or recognized by CTL [39,40]. 

### 2.2. HLA Class I as a Major Determinant of HIV-1 Pathogenesis

Rates of clinical disease progression during natural HIV-1 infection vary widely, and this has been attributed mainly to differences in HLA-restricted CTL responses [40,51,52]. The association of CTL responses with initial control of plasma viremia during primary HIV-1 infection [53,54], delayed disease progression [55] and the observation that rhesus macaques depleted of CD8+ cells prior to SIV infection exhibited an inability to control viremia [56] strongly support a critical role for CTL in control of HIV-1 at early times following infection. Over the course of infection, CTL selection pressure results in the generation of viral escape variants [53], and HLA-restricted CTL responses are a major selective force driving viral evolution in an infected host [39]. As a result of immune escape and immune dysfunction, CTL ultimately fail to control infection and the vast majority of individuals’ progress to AIDS in the absence of HAART.

HIV-1 long-term non-progressors (LTNP) and spontaneous controllers have been carefully examined in order to elucidate mechanisms of viral pathogenesis and to identify novel correlates of immune-mediated protection. It has been observed that “protective” HLA-I alleles, most notably B*27 and B*57, are consistently associated with enhanced control of HIV [57]. More recently, genome-wide association studies (GWAS) have identified single-nucleotide polymorphisms (SNPs) in the HLA region on human chromosome 6 as major determinants of lower plasma viral load set point [58,59] as well as the HIV elite controller phenotype [60,61,62]. Understanding potential differences in the ability of Nef to modulate CTL responses in the context of different HLA alleles or viral peptides is an area of research that merits further attention [45,63].

## 3. Nef-Dependent Immune Evasion: *In Vivo* and *in Vitro* Observations

*In vivo* observations have clearly suggested that Nef plays a critical role for maintenance of high plasma viremia and for progression to AIDS [9,10,11]; however, evidence to directly link Nef-mediated HLA-I down-regulation to clinical outcome is more limited. Studies using the SIV-infected macaque model system have observed that Nef mutations that impaired HLA down-regulation activity were restored during the course of infection [64,65], and it was recently shown that high plasma viremia following SIV infection correlated with a high level of *in vivo* HLA-I down-regulation [66]. Similarly, it has been reported that patient-derived Nef sequences collected during early or chronic HIV-1 infection retained significant ability to down-regulate HLA-I and to evade CTL killing [16,67]; however, other data indicate that this function may be dispensable during very late stage disease [34]. Altogether, these results indicate that HLA down-regulation is a very important *in vivo* Nef function that is maintained presumably in order to evade ongoing CTL immune pressure on the virus.

*In vitro* studies from a number of research groups have observed substantial variation in the susceptibility of HIV-infected cells to recognition and killing by CTL using standard co-culture assays [36,68,69,70]. This result is likely due to the use of different target cells, different CTL clones, and viral strains that may encode Nef variants with differential levels of expression or subtle differences in HLA down-regulation function. In cases where CTL-mediated recognition or killing has been compared directly between HIV strains that encode the *nef* gene *versus* variants harboring deletions or non-functional alleles, Nef expression has clearly been shown to confer a protective effect in both cell lines and in primary T cell assays [36,63,70,71,72]. Notably, even though Nef effectively reduces CTL-mediated killing of virus-infected cells, it may not fully abrogate the ability of responding CTL to produce antiviral chemokines or cytokines [63]. While such incomplete evasion of CTL permits non-cytolytic immune mechanisms to participate in control of chronic infection [73], it nevertheless allows HIV-1 to establish a persistent infection in the face of robust antiviral host immunity.

## 4. Nef Structure and HLA Class I Down-Regulation Function

### 4.1. General Features of Nef and its Domain Structure

Nef serves as an adaptor protein and its various functions appear to utilize non-canonical motifs and interactions to form higher-order complexes with cellular proteins [74]. Despite its small size, Nef engages with multiple and diverse host cell proteins, thereby altering their normal function to favor viral replication. Biochemical characterization of these protein complexes remains difficult, likely due to the small size of Nef, the heterogeneous nature of the complexes that are formed, and the localization of these complexes within lipid membranes.

The protein structure of Nef can be broadly divided into three domains: an N-terminal anchor and flexible loop, a central structured core, and a C-terminal flexible loop [75,76]; and two amino acid positions have been shown to be particularly critical for Nef function. The N-terminal anchor domain of Nef is required for membrane association and localization into detergent-insoluble “lipid rafts” [77], while the central core encodes numerous protein interaction and intracellular trafficking motifs that contribute differentially to diverse Nef functions [78]. The central core domain of Nef adopts a stable tertiary fold, permitting its early characterization using both NMR and X-ray crystallographic methods [79,80]. Our understanding of Nef-mediated HLA down-regulation has been significantly enhanced by the recently reported crystal structure of Nef protein in complex with the MHC-I cytoplasmic domain and the μ1 subunit of the clatherin AP1 complex [76].

### 4.2. Functional Motifs and Host Proteins Interactions

Myristoylation at glycine residue 2 (G_2_) allows the Nef protein to localize to lipid membranes, including the inner leaflet of the plasma membranes [81] and aspartic acid residue 123 (D_123_) is reported to be necessary for protein oligomerization [82]. Recent data also suggest that electrostatic interactions at D_123_ may be essential for Nef stability [76]. Site-directed mutations at either residue G_2_ or D_123_ have been shown to impair nearly all known Nef activities [82,83], however most other mutations indicate that the functional motifs required to down-regulate HLA-I are genetically separable from those required to modulate CD4. Notably, sequences in the central core domain that bind to the clatherin adaptor protein complex AP-2 [84,85], the coatomer protein ß-COP [86,87], and the vacuolar membrane ATPase V1H [88,89] are necessary for down-regulation and degradation of CD4 [90], but not HLA-I. 

The ability of Nef to down-regulate HLA-I surface expression [24,91,92] has been mapped to three distinct regions of the protein. First, an N-terminal alpha helix (R_17_ER_19_M_20_RRAEPA_26_) that contains methionine residue 20 (M_20_) serves an important as a membrane anchor [90,93], and more recently arginine residues 17 and 19 have been shown to form a second ß-COP binding motif [87]. This latter observation indicates that the final stages of Nef-mediated CD4 and HLA down-regulation may need to engage the same cellular machinery and lysosomal compartments. Second, an acidic cluster (E_62_EEE_65_) that binds to the PACS-1 and PACS-2 proteins may enhance Nef localization to the *trans*-Golgi network (TGN) and/or regulate signaling events that increase the rate of cellular endocytosis [94,95]. Finally, a polyproline (PxxP)_3_ repeat that includes proline residues 72, 75, and 78 forms an SH3-binding motif that has been shown to interact with Lyn and Hck cellular kinases [90].

### 4.3. Proposed Mechanisms of HLA Class I Down-Regulation

Two models to explain Nef-mediated HLA-I down-regulation are currently favored, including (1) altered HLA trafficking and (2) enhanced HLA internalization/turnover. These proposed mechanisms are not mutually exclusive, and indeed the two may function collaboratively within the virus-infected cell to ensure robust immune evasion.

In the ‘altered trafficking’ model, interaction between Nef and newly synthesized HLA-I occurs within the secretory pathway, disrupting normal HLA-I transport to the cell surface and redirecting it to endosome/lysosome compartments for degradation. There is strong evidence to support this model, such as the observation that Nef and HLA-I co-localize within the trans-Golgi network rather than at the cell membrane [95]. In addition, direct binding has been demonstrated between Nef and a number of cellular complexes, including AP-1 [96] and ß-COP [86]. These complexes are necessary for normal protein transport between the trans-Golgi and endosomes, and mutations in Nef that disrupt these domains dramatically impair HLA-I down-regulation function. Further support comes from biochemical data indicating that the µ1 subunit of AP-1 forms a stable interaction with Nef only in the presence of the HLA-I cytoplasmic tail [97,98,99]. The interaction between Nef and the AP-1 complex is thought to allow the µ subunit of AP-1 (which typically recognizes YxxØ motifs; where Ø is a bulky hydrophobic residue and x is any amino acid) to associate with a non-conventional sequence (Y_320_SQA_323_) in the cytoplasmic tail of HLA-I [42,99,100]. This hypothesis has been validated recently by structural determination of the Nef/µ1/HLA tripartite complex at less than 3-angstrom resolution [76], which captured this complicated interaction ‘in action’. Notably, this new structure demonstrates that a highly cooperative interaction between Nef and µ1 creates a novel binding pocket on µ1 that can accommodate a YxxA motif in HLA-I cytoplasmic tail. More specifically, Nef’s acidic cluster, E_62_EEE_65_, provides critical electrostatic interactions with µ1 that stabilize the complex and Nef’s polyproline-rich motif, PxxP, acts as a clamp to secure binding of HLA-I to µ1. These results confirm previous models based largely on biochemical data and provide clear rationale for the role of these Nef sequences in its HLA-I down-regulation activity. Furthermore, the structure by Jia *et al.* [76] highlights an important role for hydrophobic residues located in Nef’s N-terminal and C-terminal domains. While not participating directly in HLA-I or µ1 binding, Nef residues W_13_ and M_20 _anchor the protein core to the plasma membrane and presumably help to position Nef appropriately for optimal interactions with its binding partners. Likewise, residues Y_202_ and F_203_ appear to stabilize the HLA-I tail interaction.

In the ‘enhanced turnover’ model, Nef is thought to act through a series of interactions and signaling events to induce clatherin-independent internalization of HLA-I at the plasma cell membrane that requires small GTPases known as ADP-ribosylation factors (ARFs). It is proposed that Nef’s E_62_EEE_65_ motif binds to the phosphofurin acidic cluster sorting protein PACS-2 and localizes Nef within the *trans*-Golgi network (TGN) [94,95]. This allows Nef to bind to Src-family protein kinases in the TGN (in particular Hck) [101], triggering a signaling pathway that includes activation of the tyrosine kinase protein ZAP70 and PI-3-kinase, induction of phosphotidylinositol-3-phosphate (PIP_3_) on the inner leaflet of the plasma membrane, and PIP_3_-mediated recruitment of ARNO [102], that culminates in activation of ARF-6 that results in endocytosis of HLA-I [103]. Several aspects of this model remain controversial, and more recent studies have shown only modest effect of specific ARF-6 inhibitors [104] or a dominant-negative ARF6 mutant [105] on Nef-mediated HLA-I down-regulation.

The unusual functional flexibility of Nef is demonstrated by the fact that both of these HLA-I down-regulation models differ significantly from the mechanism that Nef uses to modulate CD4 expression, reviewed in [74,106]. In that case, direct interactions between Nef, the cytoplasmic tail of CD4, and the AP-2 complex at the plasma membrane [107] results in clatherin-mediated endocytosis of CD4 and its eventual degradation in lysosomes.

## 5. Natural Variation in Nef Sequence and Implications for Immune Evasion

### 5.1. Sequence Variability within Described HLA-I Down-Regulation Motifs

Nef is one of the most highly variable HIV-1 proteins; however, the impact of naturally occurring mutations on clinical outcome has rarely been explored in detail [12,108]. This is due, in part, to the fact that many of the motifs associated with its HLA-I down-regulation function are very well conserved in patient-derived sequences [12,109]. Analysis of a panel of 242 HIV-1 subtype B Nef sequence clones obtained from unique individuals by our laboratory (68 from acute infection, 122 in chronic infection, and 52 spontaneous controllers with plasma viral load <50 copies RNA/mL; *unpublished data*) confirms these previous observations (Table 1). The frequency of the consensus amino acid as well as the Shannon entropy value for each critical residue was determined using web-based tools available at the Los Alamos National Laboratory HIV Sequence Database [110]. Notably, we observed that the Nef residues required for HLA-I down-regulation function–including myristoylation (G_2_ and S_6_) and putative stability (W_13_), proline residues P_72_, P_75_, and P_78 _in the (PxxP)_3_ domain, and the aspartic acid residue D_123_–were essentially unchanged in all patient-derived sequences (all frequencies >99%). While other critical Nef residues displayed less conservation, only glutamic acid residues E_63_ and E_64_ were observed to occur in less than 90% of sequences in this cohort of individuals with broad clinical outcome. These changes were mainly conservative substitutions to aspartic acid that are expected to have modest effect on Nef function based on previous studies [111], indicating that population-level variation in the acidic domain may also be limited.

Nef sequences adjacent to known motifs tend to be more variable, but their role in Nef function remains largely unexplored. In addition to P_72_, P_75_, and P_78_, mutations at residues Q_73_, V_74_, L_76_, and R_77_ in the (PxxP)_3_ motif have been shown to alter Nef function [112,113,114]; however, each of these residues is highly conserved in our patient-derived sequences (Table 1). Greater diversity is observed in the N-terminal motif of Nef, with consensus residues R_8_, S_9_, V_10_, and V_11_ displaying less than 70% identity in our cohort (all entropy scores >1.0; *data not shown*). Similarly, we observed substantial variability in the C-terminal loop, where Y_202_ and Y_203_ displayed frequencies of ~90%. Finally, a number of Nef codons were shown recently by Lewis *et al*. [115] to be subject to strong purifying selection pressure by CTL and to impair Nef’s HLA down-regulation function, including novel polymorphisms N52A, A84D, Y135F, G140R, S169I, and V180E that displayed a range of diversity in our patient sequences. More detailed studies of these regions are warranted to fully explore the possibility that common changes in Nef sequence may affect clinical outcome. 

**Table 1:** Sequence conservation in HIV-1 negative factor (Nef) motifs required for HLA-I down-regulation.

Nef Domain ^a^	Role	AA	Frequency ^b^	Entropy ^b^	References
MG_2_xxxS_6_	Myristoylation	G_2_	100 %	0	[116]
		S_6_	99.2 %	0.05	[116]
W_13_	Stability (?)	W_13_	100 %	0	[76]
R_17_xR_19_	ß-COP	R_17_	97.5 %	0.15	[87]
		E_18_	96.7%	0.18	[115]
		R_19_	90.5 %	0.35	[87]
M_20_	Stability (?)	M_20_	90.5 %	0.37	[93]
?	Unknown	N_52_	98.8%	0.08	[115]
E_62_EEE_65_	PACS-1/2	E_62_	92.1 %	0.31	[95,115]
		E_63_	75.2 %	0.72	[95]
		E_64_	88.8 %	0.48	[95]
		E_65_	91.7 %	0.35	[95]
P_72_xxPxR_77_	SH3 binding,	P_72_	100 %	0	[76,81,117]
and (PxxP)_3_	HLA-I “clamp”	Q_73_	99.6 %	0.03	[76,81,117]
		V_74_	99.4 %	0.05	[76,81,115,117]
		P_75_	100 %	0	[76,81,117]
		L_76_	96.7 %	0.16	[76,81,117]
		R_77_	100 %	0	[76,81,117]
		P_78_	99.6 %	0.03	[76,81,117]
		G_83_	56.2%	0.73	[90,115]
?	Unknown	A_84_	99.2%	0.05	[115]
D_123_	Oligomerization and Stability (?)	D_123_	100 %	0	[74,76,82,115]
?	Unknown	Y_135_	75.2%	0.64	[115]
?	Unknown	G_140_	100%	0	[76,115]
?	Unknown	S_169_	89.7%	0.47	[76,115]
D_175_	Trafficking	D_175_	99.6%	0.03	[76,88,115,118]
?	Unknown	V_180_	99.2%	0.05	[76,115]
Y_202_	Stability (?)	Y_202_	87.6 %	0.39	[76]
F_203_^c^	Stability (?)	F_203_	9.5 %	0.31	[76]
		Y_203_	90.5 %	0.31	[76]
D_123_	Oligomerization and Stability (?)	D_123_	100 %	0	[74,76,82,115]
?	Unknown	Y_135_	75.2%	0.64	[115]
?	Unknown	G_140_	100%	0	[76,115]
?	Unknown	S_169_	89.7%	0.47	[76,115]
D_175_	Trafficking	D_175_	99.6%	0.03	[76,88,115,118]

^a^: Protein locations based on HXB2 numbering [110]; ^b^: Frequency of consensus residue and Shannon entropy score calculated using 242 clonal Nef sequences collected from unique HIV-1 subtype B-infected individuals from North America (68 acute, 122 chronic, and 52 controllers) and the Entropy-One tool (HIV Sequence Database; [110]); ^c^: Nef used by Jia *et al*. [76] encoded phenylalanine-203, but tyrosine-203 is prevalent in most sequences.

### 5.2. Immune-Mediated Attenuation of Nef Function?

HIV-1 Nef is highly targeted by the host immune response during primary infection [119], and a large number of HLA-associated polymorphisms have been identified within or near known CTL epitopes [120]. HLA-mediated immune pressure on Nef drives rapid selection of escape mutations following infection [121,122], and HLA-associated polymorphisms have been identified at approximately half of Nef’s 206 residues [120,123]. Indeed, a substantial proportion of natural sequence variation observed in Nef is attributable to immune selection pressure on this protein [120,124]. It has been presumed that the extensive ability of Nef to incorporate sequence changes would allow it to escape from immune pressure with limited consequence for viral fitness. However, while the impact of certain CTL escape mutations on Nef function has been assessed [35,125,126], the broader impact of HLA-restricted pressure on Nef function and viral pathogenesis at the individual or population level has not been clarified, and data to address this important issue are currently lacking.

We have previously reported that HLA-B*35-associated CTL escape mutations R75T and Y85F located in the conserved proline-rich region of Nef can impair HLA-I down-regulation activity [35,126]. Along with other data for A*02 [125], these observations indicate that host immune pressure can alter Nef function in at least some cases and further highlight the need for additional studies to characterize the wide array of Nef sequence variants that are likely to arise within an individual during natural infection. Indeed, population-level analyses have identified a number of HLA-associated polymorphisms in the N-terminal and C-terminal domains of Nef, as well as in sites near other critical Nef residues [120], including several that are selected by the protective allele HLA-B*57. Fully understanding the potential impact of naturally occurring HLA-associated mutations on Nef function and clinical outcome will be an important area for future study.

## 6. Conclusions

Research advances have significantly improved our understanding of the HIV-1 Nef protein and the mechanisms that it uses to effectively down-regulate HLA class I expression on the surface of infected cells. Molecular and biochemical studies have identified many of Nef’s crucial binding partners and have mapped Nef sequence motifs that are required for its function. Structural data have very recently allowed us to visualize the Nef protein in complex with HLA-I and the µ1 subunit of AP-1, validating our current models and identifying potential sites for therapeutic intervention. 

Nef-mediated evasion of host immunity is expected to contribute significantly to the establishment and maintenance of persistent HIV-1 infection. However, despite recent progress in the field to understand the cellular mechanisms of Nef-mediated HLA-I down-regulation, our knowledge of Nef’s role during HIV-1 disease progression remains poor. To fully elucidate the impact of Nef during natural infection, it will be necessary to extend our current studies of lab-adapted viral isolates to include detailed analyses of patient-derived Nef proteins. Only then will we be able to fully appreciate the consequence of Nef sequence variation on protein function and make important links to clinical outcome.

## Correction

Correction A correction was published on 5 October 2012: http://www.mdpi.com/1999-4915/4/10/2014 (PDF, 364 KB)

## References

[1] Dandekar S. (2007). Pathogenesis of HIV in the gastrointestinal tract. Curr. HIV/AIDS Rep..

[2] Guadalupe M., Reay E., Sankaran S., Prindiville T., Flamm J., McNeil A., Dandekar S. (2003). Severe CD4+ T-cell depletion in gut lymphoid tissue during primary human immunodeficiency virus type 1 infection and substantial delay in restoration following highly active antiretroviral therapy. J. Virol..

[3] Lim S.G., Condez A., Lee C.A., Johnson M.A., Elia C., Poulter L.W. (1993). Loss of mucosal CD4 lymphocytes is an early feature of HIV infection. Clin. Exp. Immunol..

[4] Lyles R.H., Munoz A., Yamashita T.E., Bazmi H., Detels R., Rinaldo C.R., Margolick J.B., Phair J.P., Mellors J.W. (2000). Natural history of human immunodeficiency virus type 1 viremia after seroconversion and proximal to AIDS in a large cohort of homosexual men. Multicenter AIDS Cohort Study. J. Infect. Dis..

[5] Sierra-Aragon S., Walter H. (2012). Targets for inhibition of HIV replication: Entry, enzyme action, release and maturation. Intervirology.

[6] Mayer K.H., Venkatesh K.K. (2010). Antiretroviral therapy as HIV prevention: Status and prospects. Am. J. Public Health.

[7] Montaner J.S., Lima V.D., Barrios R., Yip B., Wood E., Kerr T., Shannon K., Harrigan P.R., Hogg R.S., Daly P. (2010). Association of highly active antiretroviral therapy coverage, population viral load, and yearly new HIV diagnoses in British Columbia, Canada: A population-based study. Lancet.

[8] Richter S.N., Frasson I., Palu G. (2009). Strategies for inhibiting function of HIV-1 accessory proteins: A necessary route to AIDS therapy?. Curr. Med. Chem..

[9] Kestler H.W., Ringler D.J., Mori K., Panicali D.L., Sehgal P.K., Daniel M.D., Desrosiers R.C. (1991). Importance of the nef gene for maintenance of high virus loads and for development of AIDS. Cell.

[10] Deacon N.J., Tsykin A., Solomon A., Smith K., Ludford-Menting M., Hooker D.J., McPhee D.A., Greenway A.L., Ellett A., Chatfield C. (1995). Genomic structure of an attenuated quasi species of HIV-1 from a blood transfusion donor and recipients. Science.

[11] Kirchhoff F., Greenough T.C., Brettler D.B., Sullivan J.L., Desrosiers R.C. (1995). Brief report: Absence of intact nef sequences in a long-term survivor with nonprogressive HIV-1 infection. N. Engl. J. Med..

[12] Kirchhoff F., Easterbrook P.J., Douglas N., Troop M., Greenough T.C., Weber J., Carl S., Sullivan J.L., Daniels R.S. (1999). Sequence variations in human immunodeficiency virus type 1 Nef are associated with different stages of disease. J. Virol..

[13] Pushker R., Jacque J.M., Shields D.C. (2010). Meta-analysis to test the association of HIV-1 nef amino acid differences and deletions with disease progression. J. Virol..

[14] Corro G., Rocco C.A., de Candia C., Catano G., Turk G., Mangano A., Aulicino P.C., Bologna R., Sen L. (2012). Genetic and functional analysis of HIV type 1 nef gene derived from long-term nonprogressor children: Association of attenuated variants with slow progression to pediatric AIDS. AIDS Res. Hum. Retrovir..

[15] Crotti A., Neri F., Corti D., Ghezzi S., Heltai S., Baur A., Poli G., Santagostino E., Vicenzi E. (2006). Nef alleles from human immunodeficiency virus type 1-infected long-term-nonprogressor hemophiliacs with or without late disease progression are defective in enhancing virus replication and CD4 down-regulation. J. Virol..

[16] Lewis M.J., Balamurugan A., Ohno A., Kilpatrick S., Ng H.L., Yang O.O. (2008). Functional adaptation of Nef to the immune milieu of HIV-1 infection *in vivo*. J. Immunol..

[17] Zuo J., Suen J., Wong A., Lewis M., Ayub A., Belzer M., Church J., Yang O.O., Krogstad P. (2012). Functional analysis of HIV type 1 Nef gene variants from adolescent and adult survivors of perinatal infection. AIDS Res. Hum. Retrovi..

[18] Klotman M.E., Kim S., Buchbinder A., de Rossi A., Baltimore D., Wong-Staal F. (1991). Kinetics of expression of multiply spliced RNA in early human immunodeficiency virus type 1 infection of lymphocytes and monocytes. Proc. Natl. Acad. Sci. USA.

[19] Landi A., Iannucci V., Nuffel A.V., Meuwissen P., Verhasselt B. (2011). One protein to rule them all: Modulation of cell surface receptors and molecules by HIV Nef. Curr. HIV Res..

[20] Miller M.D., Warmerdam M.T., Gaston I., Greene W.C., Feinberg M.B. (1994). The human immunodeficiency virus-1 nef gene product: A positive factor for viral infection and replication in primary lymphocytes and macrophages. J. Exp. Med..

[21] Munch J., Rajan D., Schindler M., Specht A., Rucker E., Novembre F.J., Nerrienet E., Muller-Trutwin M.C., Peeters M., Hahn B.H. (2007). Nef-mediated enhancement of virion infectivity and stimulation of viral replication are fundamental properties of primate lentiviruses. J. Virol..

[22] Aiken C., Konner J., Landau N.R., Lenburg M.E., Trono D. (1994). Nef induces CD4 endocytosis: Requirement for a critical dileucine motif in the membrane-proximal CD4 cytoplasmic domain. Cell.

[23] Garcia J.V., Miller A.D. (1991). Serine phosphorylation-independent downregulation of cell-surface CD4 by nef. Nature.

[24] Greenberg M.E., Iafrate A.J., Skowronski J. (1998). The SH3 domain-binding surface and an acidic motif in HIV-1 Nef regulate trafficking of class I MHC complexes. EMBO J..

[25] Schwartz O., Marechal V., le Gall S., Lemonnier F., Heard J.M. (1996). Endocytosis of major histocompatibility complex class I molecules is induced by the HIV-1 Nef protein. Nat. Med..

[26] Iafrate A.J., Carl S., Bronson S., Stahl-Hennig C., Swigut T., Skowronski J., Kirchhoff F. (2000). Disrupting surfaces of nef required for downregulation of CD4 and for enhancement of virion infectivity attenuates simian immunodeficiency virus replication *in vivo*. J Virol.

[27] Stoddart C.A., Geleziunas R., Ferrell S., Linquist-Stepps V., Moreno M.E., Bare C., Xu W., Yonemoto W., Bresnahan P.A., McCune J.M. (2003). Human immunodeficiency virus type 1 Nef-mediated downregulation of CD4 correlates with Nef enhancement of viral pathogenesis. J. Virol..

[28] Tanaka M., Ueno T., Nakahara T., Sasaki K., Ishimoto A., Sakai H. (2003). Downregulation of CD4 is required for maintenance of viral infectivity of HIV-1. Virology.

[29] Ross T.M., Oran A.E., Cullen B.R. (1999). Inhibition of HIV-1 progeny virion release by cell-surface CD4 is relieved by expression of the viral Nef protein. Curr. Biol..

[30] Arganaraz E.R., Schindler M., Kirchhoff F., Cortes M.J., Lama J. (2003). Enhanced CD4 down-modulation by late stage HIV-1 nef alleles is associated with increased Env incorporation and viral replication. J. Biol. Chem..

[31] Benson R.E., Sanfridson A., Ottinger J.S., Doyle C., Cullen B.R. (1993). Downregulation of cell-surface CD4 expression by simian immunodeficiency virus Nef prevents viral super infection. J. Exp. Med..

[32] Michel N., Allespach I., Venzke S., Fackler O.T., Keppler O.T. (2005). The Nef protein of human immunodeficiency virus establishes superinfection immunity by a dual strategy to downregulate cell-surface CCR5 and CD4. Curr. Biol..

[33] Mwimanzi P., Hasan Z., Tokunaga M., Gatanaga H., Oka S., Ueno T. (2010). Naturally arising HIV-1 Nef variants conferring escape from cytotoxic T lymphocytes influence viral entry co-receptor expression and susceptibility to superinfection. Biochem. Biophys. Res. Commun..

[34] Carl S., Greenough T.C., Krumbiegel M., Greenberg M., Skowronski J., Sullivan J.L., Kirchhoff F. (2001). Modulation of different human immunodeficiency virus type 1 Nef functions during progression to AIDS. J. Virol..

[35] Ueno T., Motozono C., Dohki S., Mwimanzi P., Rauch S., Fackler O.T., Oka S., Takiguchi M. (2008). CTL-mediated selective pressure influences dynamic evolution and pathogenic functions of HIV-1 Nef. J. Immunol..

[36] Collins K.L., Chen B.K., Kalams S.A., Walker B.D., Baltimore D. (1998). HIV-1 Nef protein protects infected primary cells against killing by cytotoxic T lymphocytes. Nature.

[37] Leonard J.A., Filzen T., Carter C.C., Schaefer M., Collins K.L. (2011). HIV-1 Nef disrupts intracellular trafficking of major histocompatibility complex class I, CD4, CD8, and CD28 by distinct pathways that share common elements. J. Virol..

[38] Malim M.H., Emerman M. (2008). HIV-1 accessory proteins–ensuring viral survival in a hostile environment. Cell Host Microbe.

[39] Carlson J.M., Brumme Z.L. (2008). HIV evolution in response to HLA-restricted CTL selection pressures: A population-based perspective. Microbes Infect..

[40] Goulder P.J., Watkins D.I. (2004). HIV and SIV CTL escape: Implications for vaccine design. Nat. Rev. Immunol..

[41] Chen D.Y., Balamurugan A., Ng H.L., Cumberland W.G., Yang O.O. (2012). Epitope targeting and viral inoculum are determinants of Nef-mediated immune evasion of HIV-1 from cytotoxic T lymphocytes. Blood.

[42] Le Gall S., Erdtmann L., Benichou S., Berlioz-Torrent C., Liu L., Benarous R., Heard J.M., Schwartz O. (1998). Nef interacts with the mu subunit of clathrin adaptor complexes and reveals a cryptic sorting signal in MHC I molecules. Immunity.

[43] Williams M., Roeth J.F., Kasper M.R., Fleis R.I., Przybycin C.G., Collins K.L. (2002). Direct binding of human immunodeficiency virus type 1 Nef to the major histocompatibility complex class I (MHC-I) cytoplasmic tail disrupts MHC-I trafficking. J. Virol..

[44] Cohen G.B., Gandhi R.T., Davis D.M., Mandelboim O., Chen B.K., Strominger J.L., Baltimore D. (1999). The selective downregulation of class I major histocompatibility complex proteins by HIV-1 protects HIV-infected cells from NK cells. Immunity.

[45] Rajapaksa U.S., Li D., Peng Y.C., McMichael A.J., Dong T., Xu X.N. (2012). HLA-B may be more protective against HIV-1 than HLA-A because it resists negative regulatory factor (Nef) mediated down-regulation. Proc. Natl. Acad. Sci. USA.

[46] Carrington M., O’Brien S.J. (2003). The influence of HLA genotype on AIDS. Annu. Rev. Med..

[47] Jenkins M.R., Griffiths G.M. (2010). The synapse and cytolytic machinery of cytotoxic T cells. Curr. Opin. Immunol..

[48] Horst D., Verweij M.C., Davison A.J., Ressing M.E., Wiertz E.J. (2011). Viral evasion of T cell immunity: Ancient mechanisms offering new applications. Curr. Opin. Immunol..

[49] Kirchhoff F., Schindler M., Specht A., Arhel N., Munch J. (2008). Role of Nef in primate lentiviral immunopathogenesis. Cell Mol. Life Sci..

[50] Lin A., Xu H., Yan W. (2007). Modulation of HLA expression in human cytomegalovirus immune evasion. Cell Mol. Immunol..

[51] Altfeld M., Kalife E.T., Qi Y., Streeck H., Lichterfeld M., Johnston M.N., Burgett N., Swartz M.E., Yang A., Alter G. (2006). HLA alleles associated with delayed progression to AIDS contribute strongly to the initial CD8(+) T cell Response against HIV-1. PLoS Med..

[52] Kiepiela P., Leslie A.J., Honeyborne I., Ramduth D., Thobakgale C., Chetty S., Rathnavalu P., Moore C., Pfafferott K.J., Hilton L. (2004). Dominant influence of HLA-B in mediating the potential co-evolution of HIV and HLA. Nature.

[53] Borrow P., Lewicki H., Wei X., Horwitz M.S., Peffer N., Meyers H., Nelson J.A., Gairin J.E., Hahn B.H., Oldstone M.B. (1997). Antiviral pressure exerted by HIV-1-specific cytotoxic T lymphocytes (CTLs) during primary infection demonstrated by rapid selection of CTL escape virus. Nat. Med..

[54] Koup R.A., Safrit J.T., Cao Y., Andrews C.A., McLeod G., Borkowsky W., Farthing C., Ho D.D. (1994). Temporal association of cellular immune responses with the initial control of viremia in primary human immunodeficiency virus type 1 syndrome. J. Virol..

[55] Turnbull E.L., Lopes A.R., Jones N.A., Cornforth D., Newton P., Aldam D., Pellegrino P., Turner J., Williams I., Wilson C.M. (2006). HIV-1 epitope-specific CD8+ T cell responses strongly associated with delayed disease progression cross-recognize epitope variants efficiently. J. Immunol..

[56] Schmitz J.E., Kuroda M.J., Santra S., Sasseville V.G., Simon M.A., Lifton M.A., Racz P., Tenner-Racz K., Dalesandro M., Scallon B.J. (1999). Control of viremia in simian immunodeficiency virus infection by CD8+ lymphocytes. Science.

[57] Deeks S.G., Walker B.D. (2007). Human immunodeficiency virus controllers: Mechanisms of durable virus control in the absence of antiretroviral therapy. Immunity.

[58] Fellay J., Ge D., Shianna K.V., Colombo S., Ledergerber B., Cirulli E.T., Urban T.J., Zhang K., Gumbs C.E., Smith J.P. (2009). Common genetic variation and the control of HIV-1 in humans. PLoS Genet.

[59] Fellay J., Shianna K.V., Ge D., Colombo S., Ledergerber B., Weale M., Zhang K., Gumbs C., Castagna A., Cossarizza A. (2007). A whole-genome association study of major determinants for host control of HIV-1. Science.

[60] Limou S., le Clerc S., Coulonges C., Carpentier W., Dina C., Delaneau O., Labib T., Taing L., Sladek R., Deveau C. (2009). Genomewide association study of an AIDS-nonprogression cohort emphasizes the role played by HLA genes (ANRS Genomewide Association Study 02). J. Infect. Dis..

[61] McLaren P.J., Ripke S., Pelak K., Weintrob A.C., Patsopoulos N.A., Jia X., Erlich R.L., Lennon N.J., Kadie C.M., Heckerman D. (2012). Fine-mapping classical HLA variation associated with durable host control of HIV-1 infection in African Americans. Hum. Mol. Genet..

[62] Pereyra F., Jia X., McLaren P.J., Telenti A., de Bakker P.I., Walker B.D., Ripke S., Brumme C.J., Pulit S.L., Carrington M. (2010). The major genetic determinants of HIV-1 control affect HLA class I peptide presentation. Science.

[63] Tomiyama H., Akari H., Adachi A., Takiguchi M. (2002). Different effects of Nef-mediated HLA class I down-regulation on human immunodeficiency virus type 1-specific CD8(+) T-cell cytolytic activity and cytokine production. J. Virol..

[64] Munch J., Stolte N., Fuchs D., Stahl-Hennig C., Kirchhoff F. (2001). Efficient class I major histocompatibility complex down-regulation by simian immunodeficiency virus Nef is associated with a strong selective advantage in infected rhesus macaques. J. Virol..

[65] Swigut T., Alexander L., Morgan J., Lifson J., Mansfield K.G., Lang S., Johnson R.P., Skowronski J., Desrosiers R. (2004). Impact of Nef-mediated downregulation of major histocompatibility complex class I on immune response to simian immunodeficiency virus. J. Virol..

[66] Friedrich T.C., Piaskowski S.M., Leon E.J., Furlott J.R., Maness N.J., Weisgrau K.L., Mac Nair C.E., Weiler A.M., Loffredo J.T., Reynolds M.R. (2010). High viremia is associated with high levels of *in vivo* major histocompatibility complex class I Downregulation in rhesus macaques infected with simian immunodeficiency virus SIVmac239. J. Virol..

[67] Noviello C.M., Pond S.L., Lewis M.J., Richman D.D., Pillai S.K., Yang O.O., Little S.J., Smith D.M., Guatelli J.C. (2007). Maintenance of Nef-mediated modulation of major histocompatibility complex class I and CD4 after sexual transmission of human immunodeficiency virus type 1. J. Virol..

[68] Shankar P., Xu Z., Lieberman J. (1999). Viral-specific cytotoxic T lymphocytes lyse human immunodeficiency virus-infected primary T lymphocytes by the granule exocytosis pathway. Blood.

[69] Yang O.O., Kalams S.A., Rosenzweig M., Trocha A., Jones N., Koziel M., Walker B.D., Johnson R.P. (1996). Efficient lysis of human immunodeficiency virus type 1-infected cells by cytotoxic T lymphocytes. J. Virol..

[70] Yang O.O., Nguyen P.T., Kalams S.A., Dorfman T., Gottlinger H.G., Stewart S., Chen I.S., Threlkeld S., Walker B.D. (2002). Nef-mediated resistance of human immunodeficiency virus type 1 to antiviral cytotoxic T lymphocytes. J. Virol..

[71] Ali A., Ng H.L., Dagarag M.D., Yang O.O. (2005). Evasion of cytotoxic T lymphocytes is a functional constraint maintaining HIV-1 Nef expression. Eur. J. Immunol..

[72] Lewinsohn D.A., Lines R., Lewinsohn D.M., Riddell S.R., Greenberg P.D., Emerman M., Bartz S.R. (2002). HIV-1 Vpr does not inhibit CTL-mediated apoptosis of HIV-1 infected cells. Virology.

[73] Wong J.K., Strain M.C., Porrata R., Reay E., Sankaran-Walters S., Ignacio C.C., Russell T., Pillai S.K., Looney D.J., Dandekar S. (2010). *In vivo* CD8+ T-cell suppression of siv viremia is not mediated by CTL clearance of productively infected cells. PLoS Pathog..

[74] Foster J.L., Denial S.J., Temple B.R., Garcia J.V. (2011). Mechanisms of HIV-1 Nef function and intracellular signaling. J. Neuroimmune Pharmacol..

[75] Foster J.L., Garcia J.V. (2008). HIV-1 Nef: At the crossroads. Retrovirology.

[76] Jia X., Singh R., Homann S., Yang H., Guatelli J., Xiong Y. (2012). Structural basis of evasion of cellular adaptive immunity by HIV-1 Nef. Nat. Struct. Mol. Biol..

[77] Giese S.I., Woerz I., Homann S., Tibroni N., Geyer M., Fackler O.T. (2006). Specific and distinct determinants mediate membrane binding and lipid raft incorporation of HIV-1(SF2) Nef. Virology.

[78] Das S.R., Jameel S. (2005). Biology of the HIV Nef protein. Indian J. Med. Res..

[79] Franken P., Arold S., Padilla A., Bodeus M., Hoh F., Strub M.P., Boyer M., Jullien M., Benarous R., Dumas C. (1997). HIV-1 Nef protein: Purification, crystallizations, and preliminary X-ray diffraction studies. Protein Sci..

[80] Grzesiek S., Bax A., Clore G.M., Gronenborn A.M., Hu J.S., Kaufman J., Palmer I., Stahl S.J., Wingfield P.T. (1996). The solution structure of HIV-1 Nef reveals an unexpected fold and permits delineation of the binding surface for the SH3 domain of Hck tyrosine protein kinase. Nat. Struct. Biol..

[81] Fackler O.T., Luo W., Geyer M., Alberts A.S., Peterlin B.M. (1999). Activation of Vav by Nef induces cytoskeletal rearrangements and downstream effector functions. Mol. Cell.

[82] Liu L.X., Heveker N., Fackler O.T., Arold S., Le Gall S., Janvier K., Peterlin B.M., Dumas C., Schwartz O., Benichou S. (2000). Mutation of a conserved residue (D123) required for oligomerization of human immunodeficiency virus type 1 Nef protein abolishes interaction with human thioesterase and results in impairment of Nef biological functions. J. Virol..

[83] Aldrovandi G.M., Gao L., Bristol G., Zack J.A. (1998). Regions of human immunodeficiency virus type 1 nef required for function *in vivo*. J. Virol..

[84] Chaudhuri R., Mattera R., Lindwasser O.W., Robinson M.S., Bonifacino J.S. (2009). A basic patch on alpha-adaptin is required for binding of human immunodeficiency virus type 1 Nef and cooperative assembly of a CD4-Nef-AP-2 complex. J. Virol..

[85] Lindwasser O.W., Smith W.J., Chaudhuri R., Yang P., Hurley J.H., Bonifacino J.S. (2008). A diacidic motif in human immunodeficiency virus type 1 Nef is a novel determinant of binding to AP-2. J. Virol..

[86] Piguet V., Gu F., Foti M., Demaurex N., Gruenberg J., Carpentier J.L., Trono D. (1999). Nef-induced CD4 degradation: A diacidic-based motif in Nef functions as a lysosomal targeting signal through the binding of beta-COP in endosomes. Cell.

[87] Schaefer M.R., Wonderlich E.R., Roeth J.F., Leonard J.A., Collins K.L. (2008). HIV-1 Nef targets MHC-I and CD4 for degradation via a final common beta-COP-dependent pathway in T cells. PLoS Pathog..

[88] Geyer M., Yu H., Mandic R., Linnemann T., Zheng Y.H., Fackler O.T., Peterlin B.M. (2002). Subunit H of the V-ATPase binds to the medium chain of adaptor protein complex 2 and connects Nef to the endocytic machinery. J. Biol. Chem..

[89] Mandic R., Fackler O.T., Geyer M., Linnemann T., Zheng Y.H., Peterlin B.M. (2001). Negative factor from SIV binds to the catalytic subunit of the V-ATPase to internalize CD4 and to increase viral infectivity. Mol. Biol. Cell.

[90] Mangasarian A., Piguet V., Wang J.K., Chen Y.L., Trono D. (1999). Nef-induced CD4 and major histocompatibility complex class I (MHC-I) down-regulation are governed by distinct determinants: N-terminal alpha helix and proline repeat of Nef selectively regulate MHC-I trafficking. J. Virol..

[91] Greenberg M., de Tulleo L., Rapoport I., Skowronski J., Kirchhausen T. (1998). A dileucine motif in HIV-1 Nef is essential for sorting into clathrin-coated pits and for downregulation of CD4. Curr. Biol..

[92] Williams M., Roeth J.F., Kasper M.R., Filzen T.M., Collins K.L. (2005). Human immunodeficiency virus type 1 Nef domains required for disruption of major histocompatibility complex class I trafficking are also necessary for coprecipitation of Nef with HLA-A2. J. Virol..

[93] Akari H., Arold S., Fukumori T., Okazaki T., Strebel K., Adachi A. (2000). Nef-induced major histocompatibility complex class I down-regulation is functionally dissociated from its virion incorporation, enhancement of viral infectivity, and CD4 down-regulation. J. Virol..

[94] Dikeakos J.D., Thomas L., Kwon G., Elferich J., Shinde U., Thomas G. (2012). An interdomain binding site on HIV-1 Nef interacts with PACS-1 and PACS-2 on endosomes to down-regulate MHC-I. Mol. Biol. Cell.

[95] Piguet V., Wan L., Borel C., Mangasarian A., Demaurex N., Thomas G., Trono D. (2000). HIV-1 Nef protein binds to the cellular protein PACS-1 to downregulate class I major histocompatibility complexes. Nat. Cell Biol..

[96] Bresnahan P.A., Yonemoto W., Ferrell S., Williams-Herman D., Geleziunas R., Greene W.C. (1998). A dileucine motif in HIV-1 Nef acts as an internalization signal for CD4 downregulation and binds the AP-1 clathrin adaptor. Curr. Biol..

[97] Noviello C.M., Benichou S., Guatelli J.C. (2008). Cooperative binding of the class I major histocompatibility complex cytoplasmic domain and human immunodeficiency virus type 1 Nef to the endosomal AP-1 complex via its mu subunit. J. Virol..

[98] Singh R.K., Lau D., Noviello C.M., Ghosh P., Guatelli J.C. (2009). An MHC-I cytoplasmic domain/HIV-1 Nef fusion protein binds directly to the mu subunit of the AP-1 endosomal coat complex. PLoS One.

[99] Wonderlich E.R., Williams M., Collins K.L. (2008). The tyrosine binding pocket in the adaptor protein 1 (AP-1) mu1 subunit is necessary for Nef to recruit AP-1 to the major histocompatibility complex class I cytoplasmic tail. J. Biol. Chem..

[100] Roeth J.F., Williams M., Kasper M.R., Filzen T.M., Collins K.L. (2004). HIV-1 Nef disrupts MHC-I trafficking by recruiting AP-1 to the MHC-I cytoplasmic tail. J. Cell Biol..

[101] Hung C.H., Thomas L., Ruby C.E., Atkins K.M., Morris N.P., Knight Z.A., Scholz I., Barklis E., Weinberg A.D., Shokat K.M., Thomas G. (2007). HIV-1 Nef assembles a Src family kinase-ZAP-70/Syk-PI3K cascade to downregulate cell-surface MHC-I. Cell Host Microbe.

[102] Venkateswarlu K., Cullen P.J. (2000). Signalling via ADP-ribosylation factor 6 lies downstream of phosphatidylinositide 3-kinase. Biochem. J..

[103] Blagoveshchenskaya A.D., Thomas L., Feliciangeli S.F., Hung C.H., Thomas G. (2002). HIV-1 Nef downregulates MHC-I by a PACS-1- and PI3K-regulated ARF6 endocytic pathway. Cell.

[104] Larsen J.E., Massol R.H., Nieland T.J., Kirchhausen T. (2004). HIV Nef-mediated major histocompatibility complex class I down-modulation is independent of Arf6 activity. Mol. Biol. Cell.

[105] Yi L., Rosales T., Rose J.J., Chowdhury B., Knutson J.R., Venkatesan S. (2010). HIV-1 Nef binds a subpopulation of MHC-I throughout its trafficking itinerary and down-regulates MHC-I by perturbing both anterograde and retrograde trafficking. J. Biol. Chem..

[106] Lindwasser O.W., Chaudhuri R., Bonifacino J.S. (2007). Mechanisms of CD4 downregulation by the Nef and Vpu proteins of primate immunodeficiency viruses. Curr. Mol. Med..

[107] Peterlin B.M., Trono D. (2003). Hide, shield and strike back: How HIV-infected cells avoid immune eradication. Nat. Rev. Immunol..

[108] Arhel N.J., Kirchhoff F. (2009). Implications of Nef: Host cell interactions in viral persistence and progression to AIDS. Curr. Top. Microbiol. Immunol..

[109] Shugars D.C., Smith M.S., Glueck D.H., Nantermet P.V., Seillier-Moiseiwitsch F., Swanstrom R. (1993). Analysis of human immunodeficiency virus type 1 nef gene sequences present *in vivo*. J. Virol..

[110] HIV Sequence Database, Los Alamos National Laboratory. www.hiv.lanl.gov.

[111] Baugh L.L., Garcia J.V., Foster J.L. (2008). Functional characterization of the human immunodeficiency virus type 1 Nef acidic domain. J. Virol..

[112] Kuo L.S., Baugh L.L., Denial S.J., Watkins R.L., Liu M., Garcia J.V., Foster J.L.  (2012). Overlapping effector interfaces define the multiple functions of the HIV-1 Nef polyproline helix. Retrovirology.

[113] Lee C.H., Saksela K., Mirza U.A., Chait B.T., Kuriyan J. (1996). Crystal structure of the conserved core of HIV-1 Nef complexed with a Src family SH3 domain. Cell.

[114] Manninen A., Hiipakka M., Vihinen M., Lu W., Mayer B.J., Saksela K. (1998). SH3-Domain binding function of HIV-1 Nef is required for association with a PAK-related kinase. Virology.

[115] Lewis M.J., Lee P., Ng H.L., Yang O.O. (2012). Immune selection in vitro reveals human immunodeficiency virus type 1 nef sequence motifs important for its immune evasion function in vivo. J Virol.

[116] Geyer M., Munte C.E., Schorr J., Kellner R., Kalbitzer H.R. (1999). Structure of the anchor-domain of myristoylated and non-myristoylated HIV-1 Nef protein. J. Mol. Biol..

[117] Saksela K., Cheng G., Baltimore D. (1995). Proline-rich (PxxP) motifs in HIV-1 Nef bind to SH3 domains of a subset of Src kinases and are required for the enhanced growth of Nef+ viruses but not for down-regulation of CD4. EMBO J..

[118] Lu X., Yu H., Liu S.H., Brodsky F.M., Peterlin B.M. (1998). Interactions between HIV1 Nef and vacuolar ATPase facilitate the internalization of CD4. Immunity.

[119] Lichterfeld M., Yu X.G., Cohen D., Addo M.M., Malenfant J., Perkins B., Pae E., Johnston M.N., Strick D., Allen T.M. (2004). HIV-1 Nef is preferentially recognized by CD8 T cells in primary HIV-1 infection despite a relatively high degree of genetic diversity. AIDS.

[120] Brumme Z.L., John M., Carlson J.M., Brumme C.J., Chan D., Brockman M.A., Swenson L.C., Tao I., Szeto S., Rosato P. (2009). HLA-associated immune escape pathways in HIV-1 subtype B Gag, Pol and Nef proteins. PLoS One.

[121] Brumme Z.L., Brumme C.J., Carlson J., Streeck H., John M., Eichbaum Q., Block B.L., Baker B., Kadie C., Markowitz M. (2008). Marked epitope- and allele-specific differences in rates of mutation in human immunodeficiency type 1 (HIV-1) Gag, Pol, and Nef cytotoxic T-lymphocyte epitopes in acute/early HIV-1 infection. J.Virol..

[122] Dong T., Zhang Y., Xu K.Y., Yan H., James I., Peng Y., Blais M.E., Gaudieri S., Chen X., Lun W. (2011). Extensive HLA-driven viral diversity following a narrow-source HIV-1 outbreak in rural China. Blood.

[123] Brumme Z.L., Brumme C.J., Heckerman D., Korber B.T., Daniels M., Carlson J., Kadie C., Bhattacharya T., Chui C., Szinger J., Mo T., Hogg R.S., Montaner J.S., Frahm N., Brander C., Walker B.D., Harrigan P.R. (2007). Evidence of differential HLA class I-mediated viral evolution in functional and accessory/regulatory genes of HIV-1. PLoS Pathog.

[124] Carlson J.M., Listgarten J., Pfeifer N., Tan V., Kadie C., Walker B.D., Ndung’u T., Shapiro R., Frater J., Brumme Z.L., Goulder P.J., Heckerman D. (2012). Widespread impact of HLA restriction on immune control and escape pathways of HIV-1. J. Virol..

[125] Ali A., Pillai S., Ng H., Lubong R., Richman D.D., Jamieson B.D., Ding Y., McElrath M.J., Guatelli J.C., Yang O.O. (2003). Broadly increased sensitivity to cytotoxic T lymphocytes resulting from Nef epitope escape mutations. J. Immunol..

[126] Mwimanzi P., Hasan Z., Hassan R., Suzu S., Takiguchi M., Ueno T. (2011). Effects of naturally-arising HIV Nef mutations on cytotoxic T lymphocyte recognition and Nef's functionality in primary macrophages. Retrovirology.

