# Supplementary material for: Human Leukocyte Antigen (HLA) Class I Down-Regulation by Human Immunodeficiency Virus Type 1 Negative Factor (HIV-1 Nef): What Might We Learn From Natural Sequence Variants?
Source: Viruses. 2012 Sep 24;4(9):1711–30. doi: 10.3390/v4091711 (PMC3499827; doi:10.3390/v4091711)

*Supplementary Correction:*

**Brockman, M.A., *et al.*, Human Leukocyte Antigen (HLA) Class I Down-Regulation by Human Immunodeficiency Virus Type 1 Negative Factor (HIV-1 Nef): What Might We Learn From Natural Sequence Variants? *Viruses* 2012, *4*, 1711-1730**

In the original manuscript, the text in figure 1 is illegible. The correct figure should be:

**Figure 1.** Presentation of viral peptide antigens by Human Leukocyte Antigen (HLA) class I. Human immunodeficiency virus type 1 (HIV-1) proviral gene expression, including RNA transcription (**a**) and protein translation (**b**); generates functional viral proteins (**c**) as well as truncated or mis-folded proteins that are degraded by the cellular proteasome complex to form short antigenic peptides (**d**); These peptides are transported from the cytoplasm into the endoplasmic reticulum (ER) (**e**) where they can be loaded onto HLA-I molecules. Peptide/HLA complexes traffic from the ER through the Golgi and secretory vesicle (SV) network to the plasma cell membrane, where the peptide antigens are presented to circulating cytotoxic T lymphocytes (CTL) (**f**); The viral Nef protein shuttles HLA molecules located at the cell surface or within the *trans*-Golgi network into lysosomal compartments (**g**); where they are degraded. In the absence of Nef-mediated HLA down-regulation, antigen-specific CTL respond to stimulation by releasing cytotoxic molecules, including perforin and granzymes, resulting in elimination of the virus-infected cell (h).

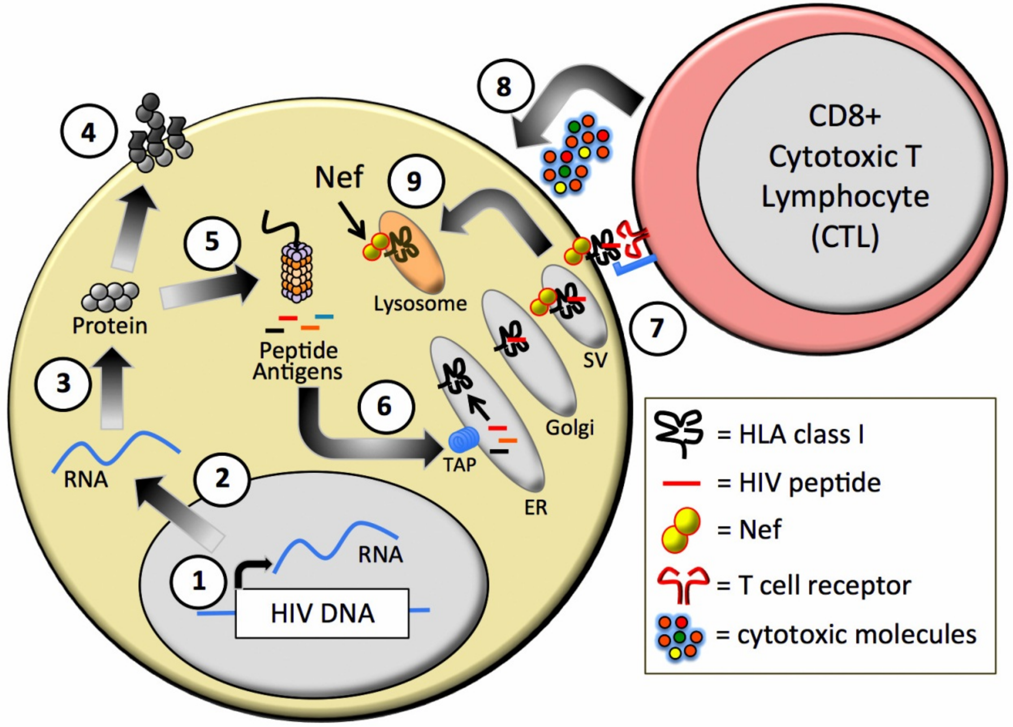

Supplement: Correction — A correction was published on 5 October 2012: http://www.mdpi.com/1999-4915/4/10/2014 (PDF, 364 KB) [file viruses-04-01711-s001.pdf]
